# Relationship between maternal–infant gut microbiota and infant food allergy

**DOI:** 10.3389/fmicb.2022.933152

**Published:** 2022-11-07

**Authors:** Shuo Wang, Rui Zhang, Xinyue Li, Yajuan Gao, Nini Dai, Yuan Wei, Luyan Liu, Yan Xing, Zailing Li

**Affiliations:** ^1^Department of Pediatrics, Peking University Third Hospital, Beijing, China; ^2^Department of Pediatrics, Fujian Provincial Maternity and Children Hospital, Fuzhou, China; ^3^Department of Obstetrics and Gynecology, Peking University Third Hospital, Beijing, China

**Keywords:** gut microbiota, early life, microbial establishment, infant, food allergy

## Abstract

The gut microbiota plays a crucial role in food allergies. We sought to identify characteristics of the maternal gut microbiota in the third trimester and the infant gut microbiota in early life and the association of these microbiotas with infant food allergy. A total of 68 healthy pregnant women and their full-term newborns were selected from a cohort of 202 mother–infant pairs; among them, 24 infants had been diagnosed with food allergy within 1 year of age, whereas 44 infants were healthy without allergic symptoms. We collected 65 maternal fecal samples before delivery and 253 infant fecal samples at five time points following birth. Fecal samples were microbiologically analyzed using 16S rRNA gene sequencing. *Holdemania* abundance in the maternal gut microbiota in the third trimester was significantly higher in the non-allergy group than in the food allergy group (*P* = 0.036). In the infant gut microbiota, *Holdemania* was only found in meconium samples; its abundance did not differ significantly between the two groups. The change in the abundance of *Actinobacteria* over time differed between the non-allergy and food allergy groups (FA, *P* = 0.013; NA, *P* = 9.8 × 10^−5^), and the change in the abundance of *Firmicutes* over time differed significantly in the non-allergy group (*P* = 0.023). The abundances of genera *Anaerotruncus, Roseburia, Ruminococcus*, and *Erysipelotricaceae* were significantly different between the non-allergy and food allergy groups at different time points. Our results showed that maternal carriage of *Holdemania* during the third trimester strongly predicted the absence of food allergies in infants; there was no correlation between the presence of food allergies and the abundance of *Holdemania* in the infant gut microbiota. More dynamic fluctuations in phyla Actinobacteria and Firmicutes early in life protect against food allergy. Thus, the enrichment of the infant gut microbiota early in life with short-chain fatty acid-producing bacteria may be beneficial in preventing the development of food allergies in infants.

## Introduction

Food allergy refers to a specific immune response that can occur repeatedly after exposure to a specific food (NIAID-Sponsored Expert Panel et al., 2010). Food allergies, especially those in infants, can increase the risk of childhood allergic diseases and reduce the quality of life of the family (Vermeulen et al., 2018; Abrams et al., 2020); thus, food allergies have emerged as a major public health problem affecting children and adults. The prevalence of food allergies has increased in recent years, affecting ~10% of the global population (Lopes and Sicherer, 2020).

The incidence and severity of food allergies have increased markedly with profound environmental and lifestyle changes, suggesting a link between an altered microbiota and the growing prevalence of allergic diseases. The hygiene hypothesis suggests that there might be a relationship between microbes and allergies (Strachan, 1989). Based on this, recent studies have proposed that antibiotic abuse, dietary changes, increased cesarean section rates, and formula feeding can alter the gut microbiota, causing an increase in the incidence of allergic diseases (Kim et al., 2019). Gut microbiota, especially in early life (0–6 months), is an important influential factor in immune and metabolic development and may have lasting consequences (Gensollen et al., 2016; Shu et al., 2019). Stable gut microbiota can promote the development of the host immune system and prevent food allergies (Chinthrajah et al., 2016; Gholizadeh et al., 2019). There is growing evidence that colonization of the gut microbiota begins in the fetus. Maternal intestinal, vaginal, and oral microbes may be the source of fetal gut microbiota, with many scholars believing that the maternal gut microbiota is the most important source (Thum et al., 2012; Hu et al., 2013; Walker et al., 2017). In a human study, pregnant women during the third trimester were administered *Lactobacillus rhamnosus*, which was discontinued at the end of pregnancy. *Lactobacillus rhamnosus* could be detected in the stool samples of babies delivered *via* vaginal delivery or cesarean section (Schultz et al., 2004), suggesting the gut microbiota in the third trimester of pregnancy influenced the colonization of the gut microbiota in infants.

To date, research on the relationship between the gut microbiota and infant disease has mainly focused on the gut microbiota during infancy; however, accumulating evidence shows that the maternal microbiome during pregnancy also plays a key role in preventing the development of an allergy-prone immune phenotype in the offspring (Gomez de Agüero et al., 2016; Vuillermin et al., 2017). Animal studies revealed that the maternal gut microbiota during pregnancy may influence the symptoms of allergic diseases in the offspring (Thorburn et al., 2015). Experiments in mice demonstrated that the maternal gut microbiota influenced neonatal adaptive immunity (Nyangahu et al., 2018). Additionally, experiments in germ-free mice have proved that the maternal gut microbiota during pregnancy affected the occurrence of allergic diseases in the offspring (Arrieta et al., 2015). Nonetheless, only one human study has found that maternal carriage of *Prevotella copri* during pregnancy reduced the risk of allergic disease in the offspring (Vuillermin et al., 2020). This suggests that human studies evaluating the relationship between maternal microbiota during pregnancy and the risk of allergic diseases in offspring are lacking.

In this study, we aimed to establish a cohort to collect and analyze the gut microbiota of pregnant women before delivery and of infants in the first 6 months after birth and investigate the relationship between maternal and infant gut microbiota and food allergy. We hypothesized that the maternal and infant gut microbiota play a role in the development of food allergy.

## Materials and methods

### Study design

This nested case–control study was conducted at the Peking University Third Hospital between February 2018 and May 2020. The study protocol was approved by the Medical Science Research Ethics Committee of Peking University Third Hospital, Peking, China (Approval No. M2018022).

### Participants

A total of 202 healthy pregnant women who underwent regular prenatal check-ups were recruited. Only the pregnant women who had regular prenatal check-ups at our hospital and were deemed by the investigator to be in good physical and mental health based on their medical history and examination were eligible to participate. The exclusion criteria were as follows: (1) underlying diseases or pregnancy complications, (2) use of antibiotics or probiotics for 2 weeks before or after delivery, and (3) refusal to participate in the study. Written consent was obtained from all participants prior to inclusion in the study. Participants who met at least one of the following criteria during the study were excluded from further participation: (1) premature birth (before 37 weeks), (2) postmature birth (after 42 weeks), (3) unstable vital signs after birth, (4) congenital malformation(s) in the infant, and (5) used of antibiotics or probiotics in infants.

### Determination of food allergy manifestations

Food allergy was diagnosed based on the following conditions: (1) one or more manifestations of poor sleep, crying, anxiety, depression, rash, runny nose, sneezing, coughing, wheezing, vomiting, diarrhea, and blood in the stool; (2) disappearance or reduction of food allergy symptoms after discontinuation of the suspected allergenic food, or reappearance or aggravation of food allergy symptoms after the reintroduction of the suspected food; (3) one or more positive results in allergen-specific IgE, food challenge test, or skin punctum test. The infant was diagnosed with food allergy by the clinician if condition 1 was present and conditions 2 and/or 3 were met.

### Clinical data and sample collection

After the establishment of the maternal–infant cohort, the health status of the mothers was investigated before delivery, and that of the infants was investigated every month after delivery until 1 year of age using a questionnaire-based survey. Stool samples were also collected. Maternal fecal samples were collected 1–2 weeks prior to the expected delivery date. Post-delivery, neonatal stool samples were collected from diapers at the following time points: first defecation; 3 days after birth; and 1, 3, and 6 months after birth. Trained professionals used sterile tubes with DNA stabilizers to collect stool samples during hospital or home visits. All samples were fully mixed with DNA stabilizer and stored at −80°C within 6 h after collection.

### DNA extraction, PCR amplification, and high-throughput sequencing

DNA was extracted from each fecal sample using the modified protocol of the QIAamp Fast DNA Stool Mini Kit (Qiagen, Hilden, Germany). Briefly, 1 ml InhibitEX buffer and glass beads (0.5-mm diameter, Qiagen) were added to a 200 mg fecal sample. The mixture was homogenized and mixed at 60 Hz for 1 min (twice) using a homogenizer. DNA was purified in accordance with the manufacturer's instructions.

The V3-V4 region of the bacterial 16S ribosomal RNA genes was amplified by PCR using barcoded primers 341F 5′-CCTACGGGRSGCAGCAG-3′ and 806R 5′-GGACTACVVGGGTATCTAATC-3′ (Wang and Qian, 2009). PCRs were performed in a volume of 30 μl containing 15 μl 2 × KAPA Library Amplification ReadyMix, 1 μl each primer (10 μM), 50 ng template DNA, and ddH_2_O (Pereira et al., 2017).

Amplicons were extracted from 2% agarose gels, purified using the AxyPrep DNA Gel Extraction Kit (Axygen Biosciences, Union City, CA, USA) according to the manufacturer's instructions, and quantified using Qubit^®^2.0 (Invitrogen, Waltham, MA USA). All quantified amplicons were pooled to equalize the concentrations for sequencing using the Illumina MiSeq PE250 (Illumina, Inc., San Diego, CA, USA). The paired-end reads (~250 bp in length) were overlapped at their 3′ ends for concatenation into original longer tags using PANDAseq (https://github.com/neufeld/pandaseq, version 2.9).

### 16S RRNA gene sequencing and statistical analysis

The lengths and average base quality of the assembled tags, trimmed barcodes, and primers were checked. 16S tags were restricted between 220 bp and 500 bp such that the average Phred score of the bases was not lower than 20 (Q20) and not higher than three ambiguous N. The copy number of the tags was enumerated, and the redundancy of repeated tags was removed. Only tags with a frequency >1, which tend to be more reliable, were clustered into operational taxonomic units (OTUs). OTUs were clustered with 97% similarity using UPARSE, and chimeric sequences were identified and removed using Usearch (Edgar, 2013). Representative OTU sequences were compared with those of the 16S rRNA genes of known species using the RDP method for classification (Cole et al., 2014).

Alpha diversity was evaluated using Chao1, Shannon, Simpson, and Observed_species indices, and the value of the alpha diversity was calculated using the QIIME (V1.9.1) software. Beta diversity was calculated using the QIIME (V1.9.1) software and an iterative algorithm with weighted and unweighted species richness information. Gplots, vegan, and ade4 packages in R were used to analyze bacterial community composition and heatmap clustering at the genus level, and the results of multivariate ANOVA based on dissimilarities (Adonis) and principal coordinates analysis (PCoA), respectively. The linear discriminant analysis effect size (LEfSe) software was used to perform LEfSe to identify potential microbial biomarkers (Segata et al., 2011).

### Statistical analysis

Statistical analyses were performed using SPSS version 25.0 (IBM, Armonk, NY, USA). Data are presented as the mean ± standard deviation, and the chi-square test of independent samples was used to analyze the differences in basic data and influencing factors between the non-allergy and allergy groups. The Wilcoxon test was used to assess the differences between the two groups at one time point. The Kruskal–Wallis test was used for analyzing the differences among periods in the non-allergy and food allergy groups. Differences were considered significant at *P* < *0.05*.

## Results

### Study participants

Of the 202 mother–infant pairs, 135 who met the criteria and were followed up regularly were included in the study cohort. They were divided into the food allergy group (FA group; *n* = 24) and the non-allergy group (NA group; *n* = 44) based on whether the infants had food allergies at 1 year of age (Supplementary Figure 1). No significant differences in maternal age, primigravida, ethnicity, and allergy symptoms were observed between the two groups (Table 1). No significant differences in the factors influencing the gut microbiota, such as delivery mode, contact with pets, number of siblings, and antibiotic consumption by the infant within 6 months of birth, and feeding factors, including breastfeeding, milk powder feeding, and time of complementary feeding, were observed between the two groups (Table 2).

**Table 1:** Clinical data of maternal participants.

**Clinical data**	**Food allergy** **(*n =* 24)**	**Non-allergy** **(*n =* 44)**	***P* ^b^**
**Age (year)^a^**	31.91 ± 2.63	32.68 ± 3.47	0.52
**Primigravida**			0.24
Yes, *n* (%)	20 (83.3)	31 (70.5)	
No, *n* (%)	4 (16.7)	13 (29.5)	
**Han ethnicity**			1.00
Yes, *n* (%)	23 (95.8)	42 (95.5)	
No, *n* (%)	1 (4.2)	2 (4.5)	
**Allergic symptoms**			0.10
Yes, *n* (%)	17(75.0)	22 (50.0)	
No, *n* (%)	7 (25.0)	22 (50.0)	

a Data are presented as the mean ± standard deviation.

b P < 0.05, indicates statistical significance.

**Table 2:** Clinical data of infant participants.

**Clinical data**	**Food allergy** **(*n =* 24)**	**Non-allergy** **(*n =* 44)**	***P* ^b^**
**Gestational age at delivery (weeks)^a^**	39.6 ± 0.89	39.9 ± 0.83	0.58
**Birth weight (g)^a^**	3.40 ± 0.17	3.52 ± 0.35	0.47
**Height (cm)^a^**	50.64 ± 2.19	50.71 ± 3.42	0.93
**Sex**			0.976
Male, *n* (%)	13 (54.2)	24 (54.5)	
Female, *n* (%)	11 (45.8)	20 (45.5)	
**Mode of delivery**			0.69
Vaginal delivery, *n* (%)	18 (75)	31 (70.5)	
Cesarean, *n* (%)	6 (25)	13 (29.5)	
**Time for complementary food**			0.341
<6 months, *n* (%)	11 (45.8)	15 (34.1)	
>6 months, *n* (%)	13 (54.2)	29 (65.9)	
**Contact with pets**			0.196
Yes, *n* (%)	7 (29.2)	7 (15.9)	
No, *n* (%)	17 (70.8)	37 (84.1)	
**Use of antibiotics**			0.699
Yes, *n* (%)	5 (20.8)	11 (25)	
No, *n* (%)	19 (79.2)	33 (75)	
**Siblings**			0.821
Yes, *n* (%)	7 (29.2)	14 (31.8)	
No, *n* (%)	17 (70.8)	30 (68.2)	
**Feeding patterns**			0.539
Breastfeeding, *n* (%)	16 (66.7)	26 (59.1)	
Mixed feeding, *n* (%)	8 (33.3)	18 (40.9)	
**Duration of breastfeeding**			0.889
<6 months, *n* (%)	3 (12.5)	5 (11.4)	
>6 months, *n* (%)	21 (87.5)	39 (88.6)	

a Data are presented as the mean ± standard deviation.

b P < 0.05, indicates statistical significance.

### Reads and OTUs

In total, 318 stool samples were collected, and the number of samples collected at different times is detailed in Supplementary Table 1. Overall, 9,020,380 high-quality reads were obtained from 253 infant samples (35,653.68±1,928.69 high-quality reads per sample, which were clustered into 2,232 OTUs). Additionally, 2,329,028 reads were obtained from 65 maternal samples (35,831±1,758.6 high-quality reads per sample, which were clustered into 1,018 OTUs).

### Differences in the change of alpha diversity of fecal microbiota in NA and FA groups

**Figure 1:**
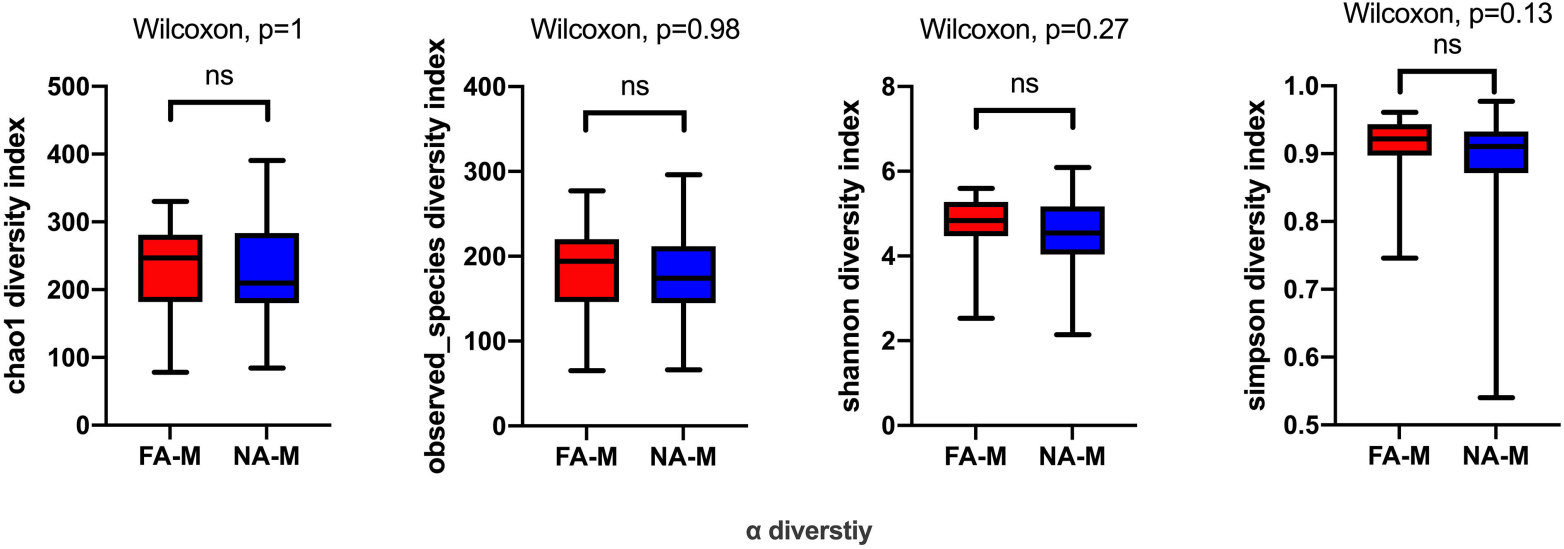
Differences in the alpha diversity of maternal samples between the non-allergy (NA) and food allergy (FA) groups, as measured by different measure indices. Chao1 and Observed_species indices reflect species richness, and Shannon or Simpson indices reflect species diversity. ns, not significant.

**Figure 2:**
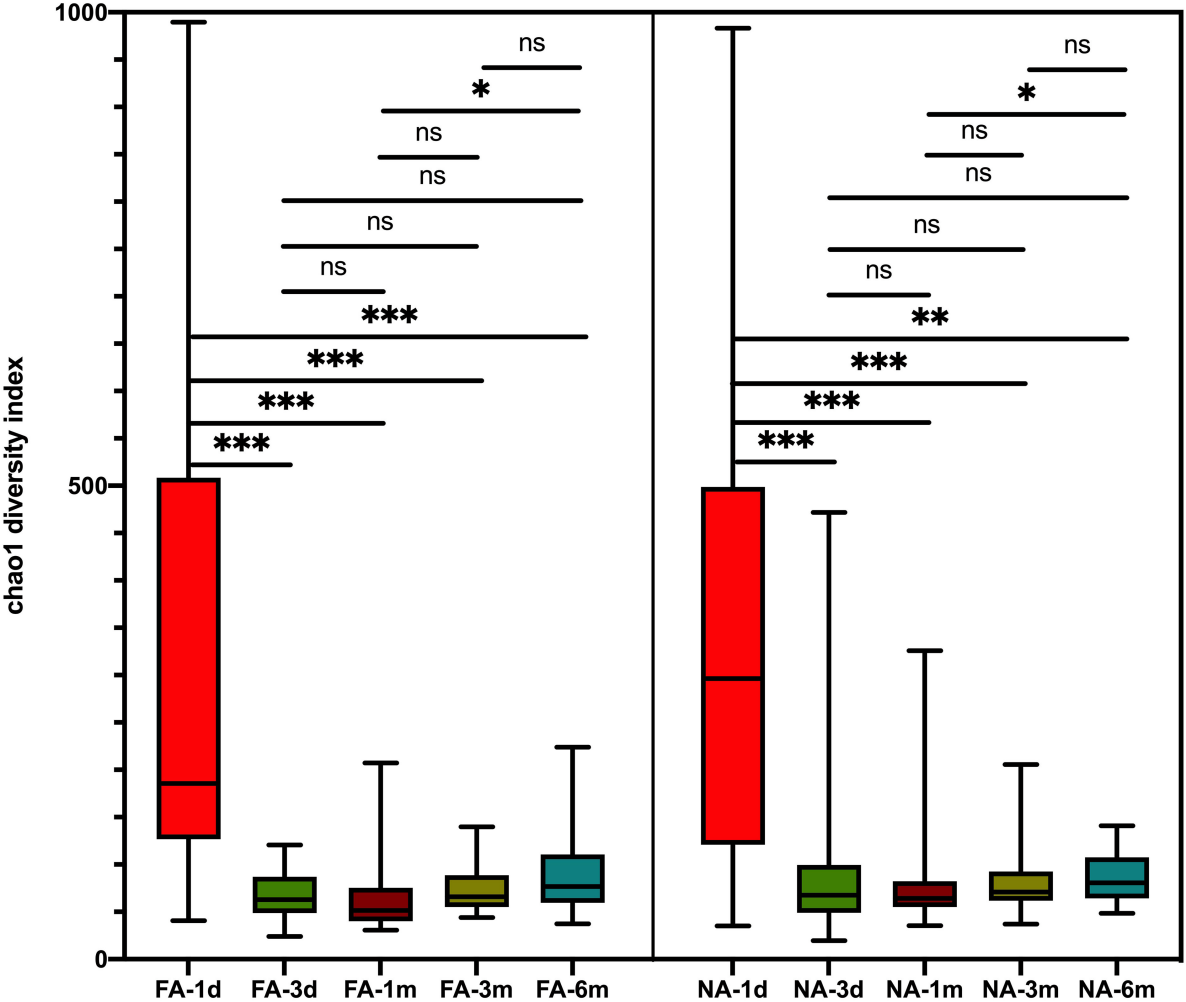
Differences in variation over time of alpha diversity between the non-allergy (NA) and food allergy (FA) groups using the Chao1 index. The Wilcoxon test was used to analyze differences among time groups in the non-allergy and food allergy groups, respectively, *P < 0.05; **P < 0.01; ***P < 0.001; ns, not significant.

In the maternal samples, although the microbial diversity in the FA group was lower than that in the NA group, neither of these indices were significantly different between the two groups (Figure 1). In the infant samples, alpha diversity indices varied over time in both the NA and FA groups and demonstrated a similar trend. In day 1 samples (meconium), richness and diversity were at a higher level than in other periods but exhibited a downward trend from day 3 to 1 month after birth. Then they continued to increase until 6 months after birth, but they did not return to their levels on day 1 (Figure 2).

**Figure 3:**
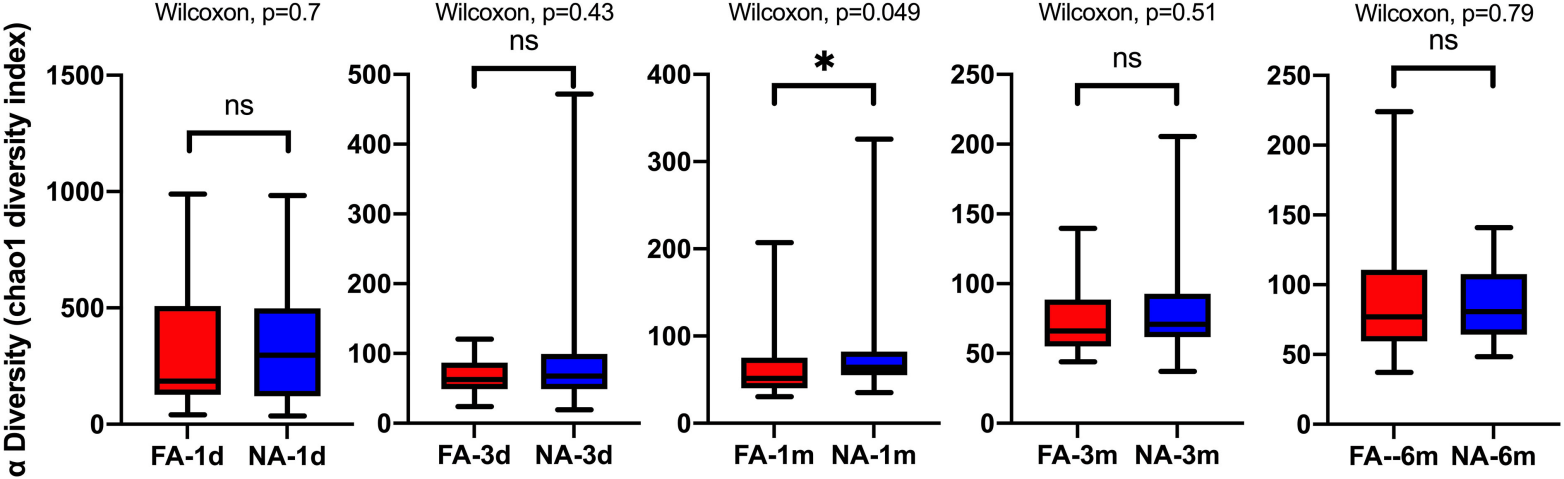
Differences in the same-time alpha diversity between non-allergy (NA) and food allergy (FA) groups using the Chao1 index. The Wilcoxon test was used to assess differences between two groups at one time point. *P < 0.05; ns, not significant.

A longitudinal comparison of the difference among different periods revealed that the NA and FA groups have similar Chao1 indices. Significant differences were observed between 1 day and other periods and between 1 month and 6 months. With respect to the Simpson, Shannon, and Observed_species indices, a comparison of every time point with day 1 showed significant differences at more time points in the NA group than in the FA group (Supplementary Figure 2). A comparison of the difference between the two groups in the same period revealed that richness and diversity in the NA group were higher than those in the FA group on day 1, day 3, 1 month, and 3 months, and there was a difference between the two groups at 1 month. At the 6-month time point, richness and diversity in the NA group were slightly lower than those in the FA group without statistical significance (Figure 3).

### Differences in the change of beta diversity of fecal microbiota in NA and FA groups

**Figure 4:**
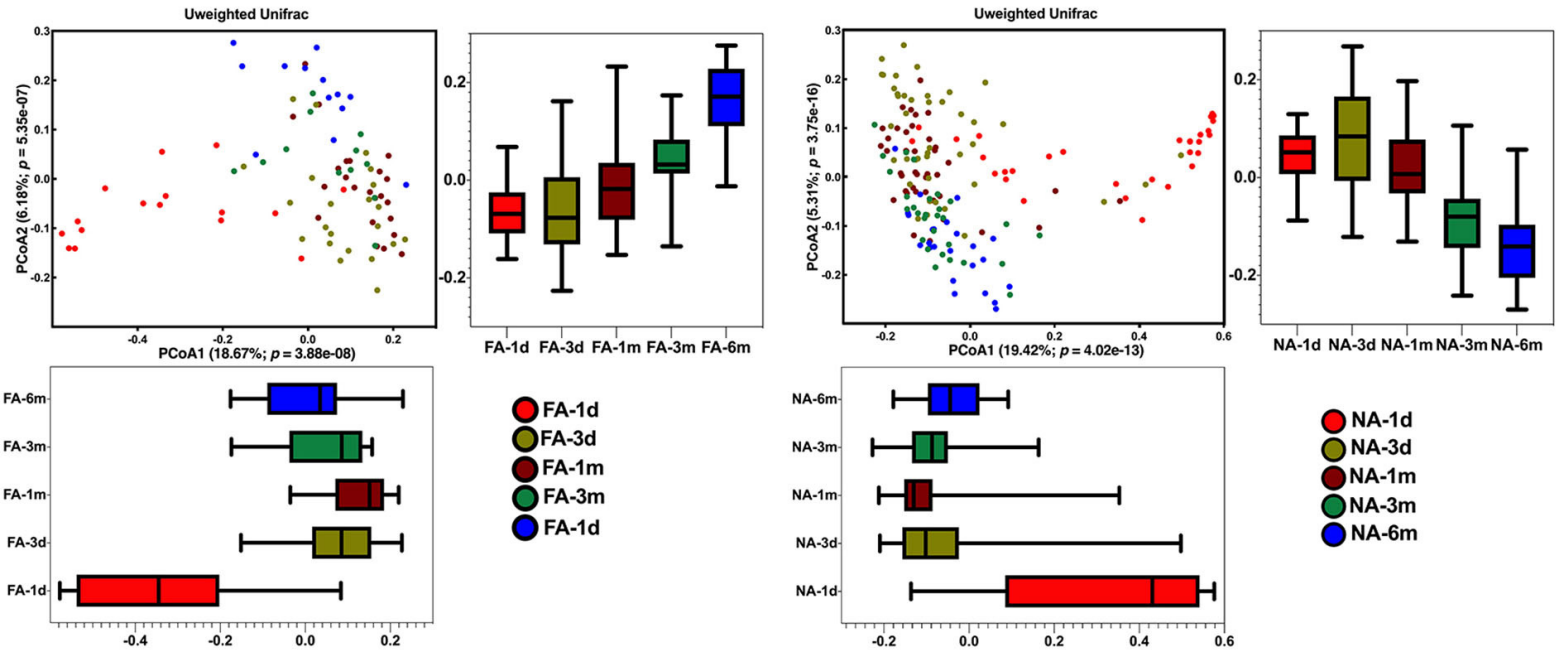
Differences in beta diversity over time between non-allergy (NA) and food allergy (FA) groups, as determined using principal coordinates analysis (PCoA) combined with Adonis analysis. *P* < 0.05 indicates a statistical significance.

Principal coordinates analysis revealed that the maternal samples between the two groups were close and the Adonis test revealed a *P*-value > 0.05, i.e., the differences were not significant (Supplementary Figure 3A). In the infant samples, a comparison of the differences in the same group at different periods showed similar results, with day 1 samples in both groups significantly clustered away from samples at other periods in the same group; the Adonis test revealed that the difference was significant (Figure 4). Comparison of the difference between the two groups in the same period showed that the NA and FA samples were tightly clustered and could not be distinguished, and the Adonis test revealed that the five indices between the two groups did not exhibit any significant differences at any time point (Supplementary Figures 3B–F).

### The composition of fecal microbiota at the phylum level varied over time in the two groups

**Figure 5:**
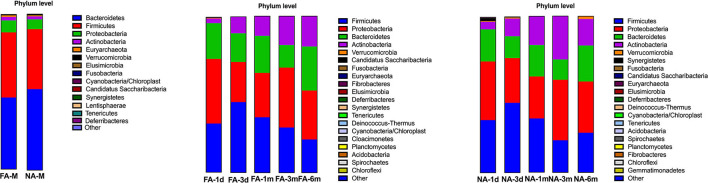
Bacterial phyla in the maternal and infant cohorts in the non-allergy (NA) and food allergy (FA) groups.

**Figure 6:**
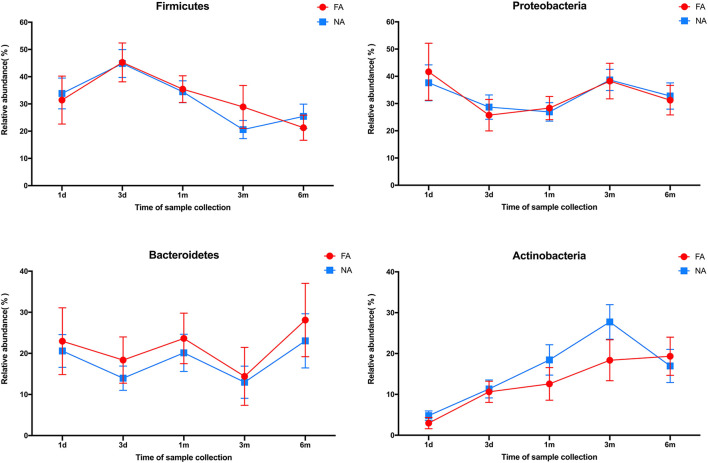
Differences in the relative abundance of major phyla over time between the non-allergy (NA) and food allergy (FA) groups.

In all samples, the gut microbiota at the phylum level was mainly enriched in Firmicutes, Actinobacteria, Proteobacteria, and Bacteroidetes. The relative abundance of Bacteroidetes (FA 46.29%, NA 51.94%) and Firmicutes (FA 42.09%, NA 38.44%) were higher in the maternal samples, and the FA and NA groups exhibited a similar relative abundance of these bacteria. The relative abundance of Proteobacteria and Firmicutes was higher in the infant samples, and the abundance of each phylum changed over time (Figure 5). The relative abundance of Proteobacteria and Bacteroidetes in the FA and NA groups did not differ over time; however, there was a difference in the abundance of Actinobacteria (at various time points in the FA and NA groups) (FA, *P* = 0.013; NA, *P* = 9.8 × 10^−5^) and Firmicutes (at various time points in the NA group) (*P* = 0.023). Actinobacteria abundance was similar on day 1 (FA, 2.97%; NA, 4.81%) and day 3 (FA, 10.62%; NA, 11.32%) and gradually increased at 1 month (FA, 12.57%; NA, 18.43%) and 3 months (FA, 18.36%; NA, 27.74%). However, at 6 months (FA 19.32%, NA 16.96%), Actinobacteria abundance in the NA group suddenly decreased, whereas that in the FA group continued to increase steadily. Firmicutes abundance in both groups presented a similar trend from day 1 to 3 months, gradually increasing from day 1 (FA, 31.46%; NA, 33.90%) to day 3 (FA, 45.26%; NA, 44.83%). After reaching the highest value on day 3, Firmicutes abundance exhibited a decreasing trend (1 month [FA, 35.43%; NA, 34.49%) and 3 months (FA, 28.90%; NA, 20.59%)]. At 6 months (FA, 21.27%; NA, 25.45%), Firmicutes abundance in the NA group suddenly increased; however, that of Firmicutes in the FA group continued to decrease steadily (Figure 6).

### The composition of fecal microbiota at the genus level varied over time in the two groups

**Figure 7:**
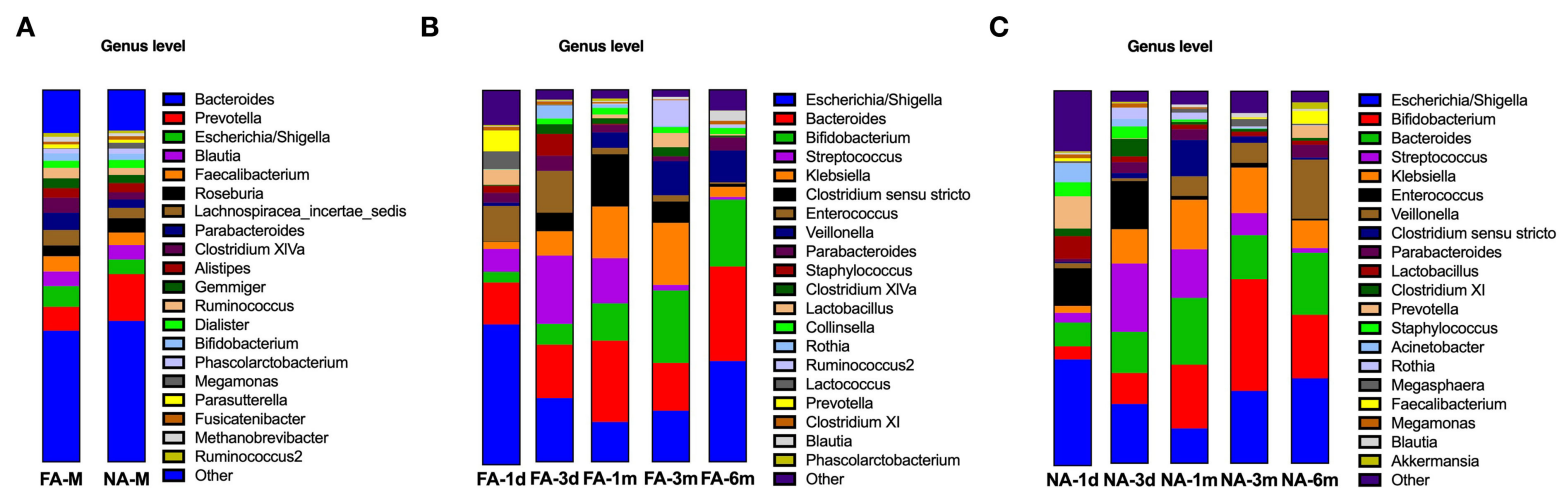
Bacterial genera in maternal **(A)** and infant **(B,C)** participants in the non-allergy (NA) and food allergy (FA) groups.

**Figure 8:**
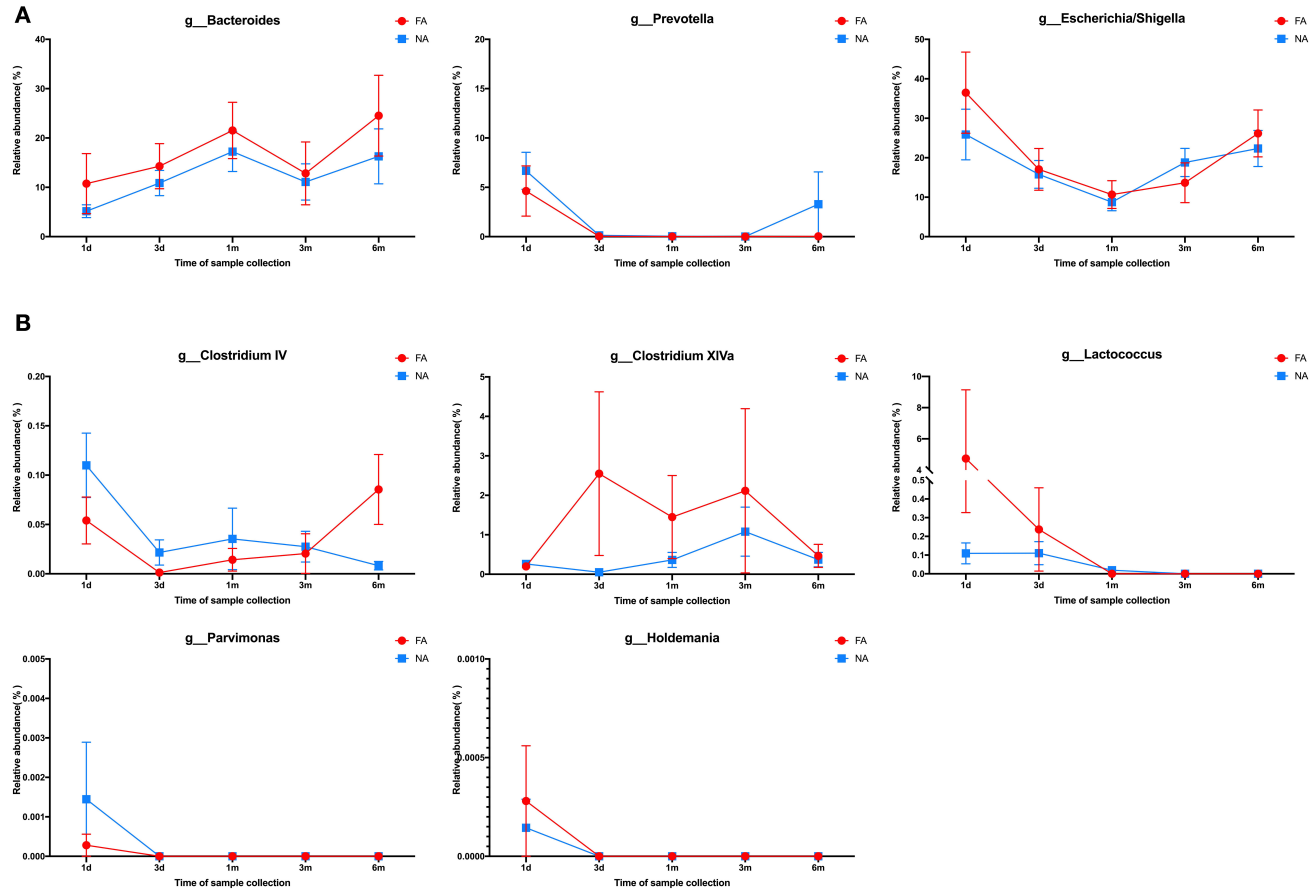
**(A)** Changes in the maternal main gut microbiota in late pregnancy compared with the infant gut microbiota over time. **(B)** Changes in the maternal differential gut microbiota in late pregnancy compared with infant gut microbiota over time.

In the maternal samples, at the genus level, among the top 20 species, *Bacteroides* accounted for the highest proportion (FA, 35.28%; NA, 37.88%), which was higher in the NA group than in the FA group, followed by *Prevotella* (FA, 6.41%; NA, 12.59%), whose proportion was higher in the NA group, and *Escherichia/Shigella* (FA, 5.56%; NA, 3.94%), whose proportion was higher in the FA group. The proportions of *Clostridium XIVa* (FA, 4.04%; NA, 1.96%), *Faecalibacterium* (FA, 4.11%; NA, 3.37%), *Parabacteroides* (FA, 4.63%; NA, 2.25%), and *Ruminococcus* (FA, 2.83%; NA, 1.89%) were higher in the FA group than in the NA group, whereas those of *Roseburia* (FA, 2.90%; NA, 3.86%), *Dialister* (FA, 1.90%; NA, 2.16%), and *Megamonas* (FA, 0.14%; NA, 1.59%) were higher in the NA group (Figure 7A). Further analysis of the abundance of the main maternal gut microbiota in the offspring's gut microbiota revealed that the proportions of *Bacteroides* and *Escherichia/Shigella* exhibited the same trend between the two groups. The abundance of *Prevotella* in the infant gut microbiota was high only on day 1 (FA, 5.58%; NA, 8.62%), low at other periods, and increased again at 6 months in the NA group (3.29%) (Figure 8A).

In the infant samples, taxonomy-based analysis revealed that the fecal microbiota in the early life (0–6 months) primarily comprised species belonging to the genera *Bacteroides* and *Escherichia/Shigella*, followed by *Bifidobacterium* and *Streptococcus*. The abundance of each genus at different periods after birth varied with respect to increase and decrease; compared with the FA group, variations in the NA group had different trends (Figures 7B,C).

The abundances of these four dominant bacterial genera varied with time and exhibited a similar trend between the NA and FA groups. *Bacteroides* had the highest proportion, and its abundance gradually increased within 1 month after birth (FA, 21.9%; NA, 17.9%), decreased at 3 months (FA, 12.9%; NA, 11.8%), and increased again at 6 months (FA, 25.4%; NA, 16.7%). The proportion of *Escherichia/Shigella* was 28.3% and 37.5% on day 1, gradually decreased to 9.3% and 10.7% at 1 month, and gradually increased to 22.8% and 27.1% at 6 months in the NA and FA groups, respectively. The proportion of *Streptococcus* in the NA group (2.6%) was lower than that in the FA group (6.1%) on day 1 and reached its highest value on day 3 with similar values in the NA (18.358%) and FA groups (18.364%). Subsequently, *Streptococcus* proportion decreased gradually and was higher at 1 month (FA, 12.13%; NA, 13.09%), 3 months (FA, 1.49%; NA, 5.91%), and 6 months (FA, 0.73%; NA, 1.26%) in the NA group than in the FA group. The proportion of *Bifidobacterium* increased gradually over time, which was different from that observed for other dominant bacterial genera. The *Bifidobacterium* proportion was the lowest on day 1 and was higher in the NA group (3.5%) than that in the FA group (2.9%). It increased gradually and reached its highest value on day 3 (FA, 5.61%; NA, 8.33%) and at 1 month (FA, 10.04%; NA, 17.17%), and was higher in the NA group (30%) than in the FA group (19.5%). At 6 months, the *Bifidobacterium* proportion returned to the same level (FA, 17.98%; NA, 17.08%).

### Comparison of gut microbiota between the NA and FA groups

**Figure 9:**
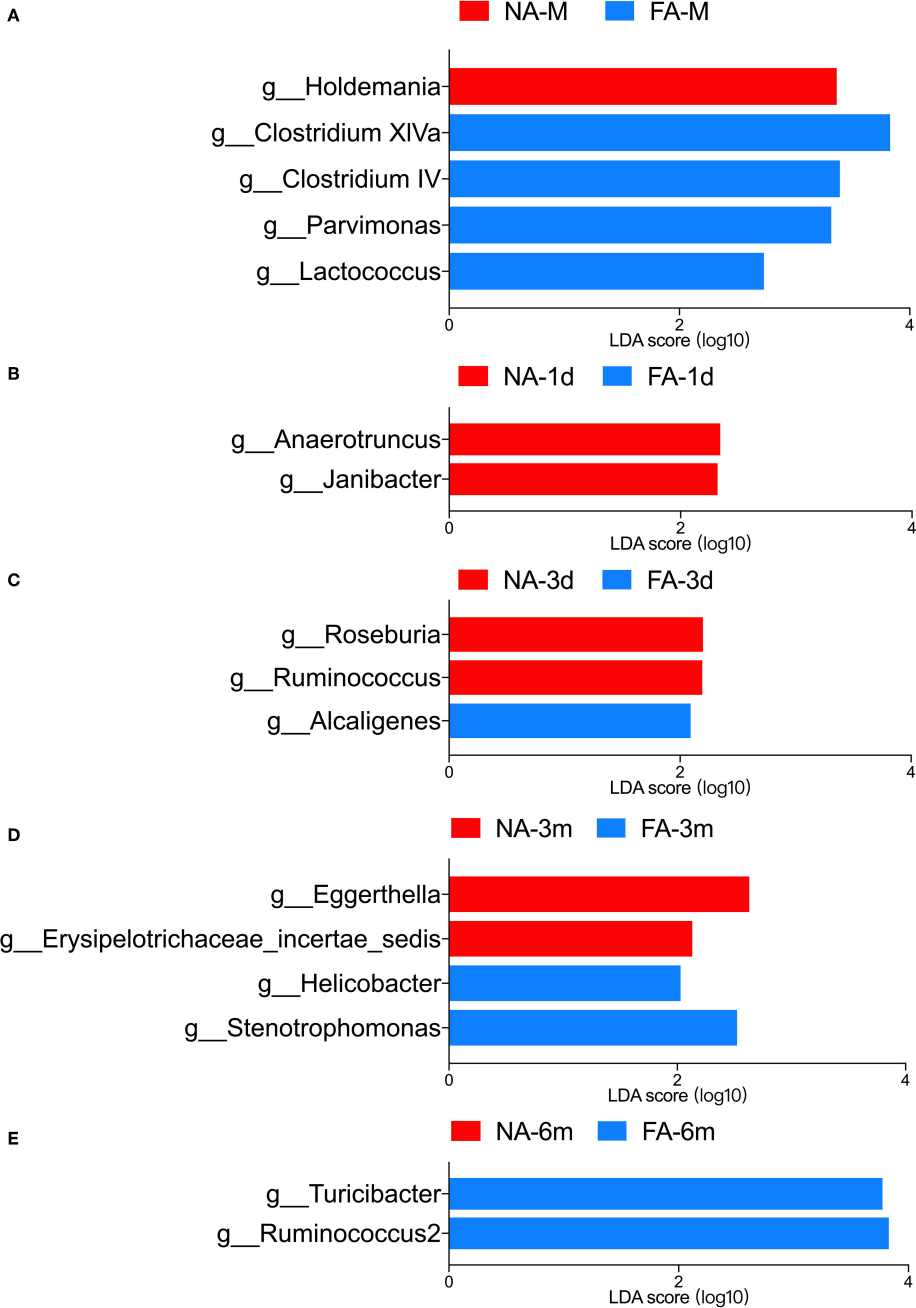
LEfSe analysis of maternal **(A)** and infant gut microbiota at different timepoints **(B–E)** was compared between the non-allergy group (NA) and food allergy (FA) groups. Only the genera with linear discriminant analysis (LDA) scores > 2 and *P* < 0.05 are presented.

LEfSe analysis was used to identify the communities or species that had a significant influence on sample division, as presented in Figure 9. There were no significant differences between the two groups at the same time points. At the genus level, significant differences in relative abundance between the two groups at the same time point are listed in Table 3.

**Table 3:** Comparison of gut microbiota at the genus level between the non-allergy and food allergy groups.

**Participants/Time**		**Genus**	**Food allergy** **(mean)**	**Non-allergy** **(mean)**	***P* ^a^**
**Mother**		*Clostridium IV*	7.98E-03	4.48E-03	0.032
		*Clostridium XlVa*	3.41E-02	1.74E-02	0.013
		*Holdemania*	7.80E-06	2.45E-05	0.036
		*Lactococcus*	8.77E-05	1.39E-05	0.033
		*Parvimonas*	2.73E-05	0	0.018
**Infant**	**Day 1**	*Acidaminococcus*	1.12E-05	0	0.015
		*Anaerotruncus*	1.12E-05	4.55E-04	0.029
		*Hydrogenophaga*	5.32E-05	1.45E-06	0.020
		*Janibacter*	1.57E-04	5.09E-04	0.018
	**Day 3**	*Alcaligenes*	6.11E-05	0	0.016
		*Roseburia*	6.93E-05	3.43E-04	0.040
		*Ruminococcus*	1.83E-05	1.34E-04	0.039
	**3 months**	*Atopobium*	5.23E-05	1.89E-04	0.016
		*Eggerthella*	9.71E-05	9.51E-04	0.021
		*Erysipelotrichaceae*	0	2.98E-04	0.049
		*Helicobacter*	3.74E-05	3.09E-06	0.0075
		*Parascardovia*	2.24E-05	0	0.029
		*Stenotrophomonas*	8.59E-05	1.55E-06	0.034
	**6 months**	*Ruminococcus2*	7.76E-03	9.34E-06	0.0016
		*Turicibacter*	9.65E-04	0	0.017

Values are expressed as the mean of the relative abundance. Only the genera with significant differences are listed.

a P <0.05 indicates statistical significance.

In the maternal samples, the relative abundance of taxa belonging to the genera *Clostridium IV* (*P* = 0.032), *Clostridium XlVa* (*P* = 0.013), *Lactococcus* (*P* = 0.033), and *Parvimonas* (*P* = 0.018) in the FA group was higher than that in the NA group. The relative abundance of *Holdemania* (*P* = 0.036) in the FA group was lower than that in the NA group. However, in the infant samples, *Holdemania* was observed only in the day-1 samples (meconium) rather than in samples of other periods, and its abundance did not differ between the two groups. Similarly, the relative abundance of *Lactococcus* in the day-1 samples was higher in the FA group than in the NA group. There was a significant decrease in abundance over time in the FA group, and in the 1, 3, and 6-month samples, the abundance of *Lactococcus* was higher in the NA group; however, there was no significant difference between the two groups. Further analysis of the changes in the different infant gut microbiota revealed that *Parvimonas* and *Holdemania* were only observed in meconium microbiota, while *Lactococcus* was only observed on day 1, day 3, and 1 month; however, there was no significant difference between the two groups. The relative abundance of *Clostridium XlVa* was the lowest on day 1, which increased and decreased over time. Altogether, the relative abundance of *Clostridium XlVa* in the FA group was greater than that in the NA group; however, there was no significant difference between the two groups at each time point. For the genus *Clostridium IV*, the relative abundance in the FA group was higher than that in the NA group at all time points except at 6 months (Figure 8B).

Comparison of the infant samples of two groups at the same time points revealed that in 1-day samples (meconium), the relative abundance of taxa belonging to the genera *Anaerotruncus* (*P* = 0.029) and *Janibacter* (*P* = 0.018) in the healthy group was higher than that in the diseased group, whereas the relative abundance of the genera *Acidaminococcus* (*P* = 0.015) and Hydrogenophaga (*P* = 0.020) in the NA group was lower than that in the FA group. In the 3-day samples, the relative abundance of the genera *Roseburia* (*P* = 0.040) and *Ruminococcus* (*P* = 0.039) in the NA group was higher than that in the FA group, and the relative abundance of genus *Alcaligenes* (*P* = 0.016) in the NA group was lower than that in the FA group. However, at 1 month, no significant difference was observed between both groups. In 3-month samples, the relative abundance of the genera *Atopobium* (*P* = 0.016), *Eggerthella* (*P* = 0.021), and *Erysipelotrichaceae* (*P* = 0.049) in the NA group was higher than that in the FA group. In 6-month samples, the relative abundance of the genus Ruminococcus2 (*P* = 0.0016) in the NA group was significantly lower than that in the FA group.

## Discussion

Accumulating evidence has demonstrated that the infant gut microbiota can influence the development of food allergies. Additionally, the influence of the maternal gut microbiota in the late trimester on the development of allergic diseases in the offspring has been proposed recently. Therefore, in this prospective study of 68 pairs of mothers and infants in China, we sought to investigate the relationship between food allergy and gut microbiota in the third trimester and early infancy by collecting and analyzing stool samples from mothers within 1–2 weeks before delivery and infants at five time points.

In this study, based on questionnaire-based survey results, the history of food allergy in the mother increased the risk of food allergy in the offspring. The maternal fecal microbiota investigated before delivery exhibited no difference in alpha diversity and beta diversity between the NA and FA groups. This suggests that the gut microbiota of healthy pregnant women in the third trimester of pregnancy is similar in species composition and abundance. However, we identified some bacterial genera whose relative abundance differed significantly between the two groups. The relative abundance of *Holdemania* was significantly higher in the NA group than in the FA group. *Holdemania* is a gram-positive anaerobic genus belonging to the family *Erysipelotrichaceae* and is involved in the catabolism of mucin, which can promote intestinal barrier damage and trigger a systemic inflammatory response after entering the intestinal mucosa (Raimondi et al., 2021). It was associated with diet during pregnancy i.e., high fiber intake was associated with a higher abundance of *Holdemania* (Gomez-Arango et al., 2018). The abundance of *Holdemania* also positively correlated with total polyunsaturated fatty acids (PUFAs) and both ω-3 and ω-6 PUFAs (Barrett et al., 2018). Meta-analysis results suggested the benefits of increased ω-3 PUFAs in the maternal diet and outcomes of childhood allergic diseases, including food allergy (Best et al., 2016). Thus, the abundance of *Holdemania* in the maternal feces during pregnancy is potentially an important predictor of food allergies in the offspring. The relative abundance of this bacterium in the infant gut microbiota was very low, suggesting that its effect on food allergy in the offspring is not correlated with its content in the infant gut microbiota. We also detected *Holdmania* only on day 1 in the infant gut microbiota, albeit not at other times. There may be a correlation between the gut microbiota of infants (meconium) and that of mothers in the third trimester.

By contrast, in the maternal samples, the relative abundance of *Clostridium IV, Clostridium XIVa*, and *Lactococcus* in the FA group was high. These *Clostridium* spp. have rarely been mentioned in studies on maternal gut microbiota and offspring food allergy; however, a concordance was observed in infant gut microbiota and allergic disease studies (Vael et al., 2011; Nylund et al., 2013; Chen et al., 2016). Importantly, the abundance of *Clostridium XIVa* and *Lactococcus* in the gut microbiota of infants with food allergies was low (Atarashi et al., 2011; Savage et al., 2018). *Lactococcus* is a probiotic that helps prevent the development of food allergies (Frossard et al., 2007; Shin et al., 2018), which is contrary to our maternal gut microbiota findings. In the infant gut microbiota, the relative abundance of this bacterium was considerably low after 1 month, and there was no significant difference between the two groups at each time point, suggesting that the effect of *Lactococcus* on infant food allergy may play a central role in the colonization of the infant's intestine.

The infant gut microbiota changes dynamically early in life. Our study revealed that gut microbiota richness in both the NA and FA groups was the highest on day 1 after birth, gradually declined thereafter, reached the lowest level at 1 month after birth, and gradually increased, with significant differences between 1 day and the other time points, suggesting that the meconium microbiota of infants may be affected by the gut microbiota in the third trimester of pregnancy. Comparison of the fecal microbiota of infants between the two groups revealed a significant difference in alpha diversity at 1 month; however, there was no difference in beta diversity at any time point. These results indicate that the gut microbiota in infants may be reconstructed after birth. This reconstruction process is divided into two stages, i.e., birth to 1 month and 1-6 months.

The gut microbiota of healthy infants also underwent dynamic reconstruction, as evidenced by changes at the phylum level. In our study, the relative abundance of Actinobacteria was similar between the two groups during birth until 1 month, with that of the NA group being slightly higher than that of the FA group. However, from 1 to 6 months, this difference in the relative abundance of Actinobacteria between the two groups gradually increased. At 6 months, Actinobacteria abundance suddenly decreased in the NA group; no such trends were observed in the FA group. Similarly, the variation trend and relative abundance of Firmicutes were similar during birth until 1 month. At 1-6 months, the difference between the two groups gradually increased. At 6 months, abundance suddenly increased in the NA group, and no such fluctuation was detected in the FA group. These results suggest that the abundance of Actinobacteria and Firmicutes fluctuated more dynamically early in life (6 months after birth), and these fluctuations may be beneficial for the prevention of food allergy. This is similar to some of the findings reported in another study (Shen et al., 2019).

We observed some differences between the NA and FA groups at the genus level. The relative abundance of *Anaerotruncus* on day 1, *Roseburia* and *Ruminococcus* on day 3, and *Erysipelotrichaceae* at 3 months were significantly higher in the NA group than in the FA group, suggesting that these genera may have a protective effect on infant food allergy. *Anaerotruncus*, belonging to the Ruminococcaceae family that includes short-chain fatty acid (SCFA)-producing bacteria (Yan et al., 2019), confers protective effects against food allergy (Shu et al., 2019). *Roseburia* is a butyrate-generating microorganism (Duncan et al., 2004; Zhuang et al., 2019), and it has been considered a potential indicator of intestinal health (Tamanai-Shacoori et al., 2017); a link has been established between *Roseburia* and intestinal diseases, including inflammatory bowel disease (Kellermayer, 2019; Luo et al., 2019; Yang et al., 2020), IBS (Chassard et al., 2012), and colon cancer (Wang et al., 2012). *Roseburia* has also been reported to strengthen intestinal barrier function and enhance Treg population expansion (Patterson et al., 2017; Yan et al., 2019), which may be beneficial with respect to food allergies. The genus *Ruminococcus* has been implicated in mediating protection from asthma through the production of SCFAs and volatile substances with the ability to reduce T-helper cell type 2-mediated allergic airway inflammation (Ege, 2017; Zhuang et al., 2019), and it has also been suggested that it may be involved in the development of ovalbumin tolerance (Xu et al., 2022). *Erysipelotrichaceae* has also been reported to be a key butyrate-producing bacterium (Estaki et al., 2016), which is reported to be more abundant in the gut microbiota during the first month of life in infants with IgE-mediated food allergy (Joseph et al., 2022). We observed that these bacteria have one thing in common: They are SCFA-producing bacteria. Recently, SCFAs, the main class of gut microbiota-derived metabolites, have been proposed to have beneficial effects with respect to food allergy. Some studies suggest that SCFA butyrate directly affects mast cells by epigenetically regulating FcεRI-mediated signaling molecules (Folkerts et al., 2018, 2020; Wang et al., 2018). Thus, by directly inhibiting IgE-mediated mast cell degranulation and allergen-induced histamine release, microbial SCFAs, such as butyrate, may exhibit therapeutic benefits with respect to human food allergies. One study reported that children with a milk allergy had lower levels of butyrate in their feces than healthy controls (Berni Canani et al., 2018). Altogether, these results establish an important role for dietary fiber and SCFAs in reinforcing the integrity of the epithelial barrier, oral tolerance, and protection against food allergy (Luu et al., 2020). Interestingly, in this study, we observed that the abundance of SCFA-producing bacteria in the feces of non-allergic infants was significantly higher than that in infants with food allergies. This suggests that a high abundance of SCFA-producing bacteria in the gut may have a protective effect with respect to food allergies, possibly *via* increased concentration of SCFAs, especially butyric acid, in the gut. However, it is necessary to further study the specific mechanisms by which gut microbiota influences the development of food allergies using mouse models.

We observed that *Prevotella* was the second dominant bacterial genus in the maternal gut microbiota. However, in the infant gut microbiota, the relative abundance of *Prevotella* was higher only on the first day after birth and lower at later time points, suggesting that the maternal gut microbiota in the third trimester of pregnancy partly affects the meconium microbiota of the offspring. Nonetheless, with the reconstruction of gut microbiota in the offspring, this effect was not retained. An increase in *Prevotella* abundance in the maternal gut microbiota during pregnancy had a protective effect on food allergy, which is unrelated to *Prevotella* abundance in the gut microbiota of the offspring (Vuillermin et al., 2020). In our study, the relative abundance of *Prevotella* in the maternal gut microbiota in the NA group was higher than that in the FA group, albeit there was no significant difference between the two groups. In both groups, the presence of *Holdemania*—associated with food allergy in the offspring—was not related to the abundance of this bacteria in the infant gut microbiota. We can further speculate that the effect of the maternal gut microbiota on food allergy in the offspring is not only mediated by regulating the changes in the infant gut microbiota but rather through metabolites.

This study had certain limitations. First, to analyze the relationship between the maternal (during pregnancy) and infant intestinal microbiota, we compared changes in the main flora and differential bacteria between the maternal (during pregnancy) and infant intestinal flora only. Second, both milk microbiota and saliva microbiota may affect the composition of the gut microbiota (Pannaraj et al., 2017; Reddel et al., 2022); however, we did not collect milk and saliva samples, which may explain the discrepancies between our results and the results of previous studies.

## Conclusion

In this study, maternal carriage of *Holdemania* during the third trimester strongly predicts the absence of food allergies in the offspring. However, it was not observed to be significantly correlated with the distribution of the gut microbiota in the progeny. The maternal gut microbiota in the third trimester of pregnancy was correlated with the infant gut microbiota on day 1 after birth (meconium). However, this effect was not retained post-reconstruction of the infant gut microbiota after birth, suggesting that the effect of maternal gut microbiota on food allergy in the offspring may not be primarily mediated through the regulation of changes in the infant gut microbiota. The infant gut microbiota undergoes dynamic changes early in life. The abundance of phyla Actinobacteria and Firmicutes fluctuates more dynamically during the early period of life, which may be beneficial for the prevention of food allergies. SCFA-producing bacteria, especially butyric acid-producing bacteria, including *Anaerotruncus, Roseburia, Ruminococcus*, and *Erysipelotricaceae*, may have a protective effect against food allergy. Nonetheless, the specific mechanism responsible for this phenomenon needs to be studied further.

## Data availability statement

The datasets presented in this study can be found in online repositories. The names of the repository/repositories and accession number(s) can be found below: NCBI - PRJNA848136.

## Ethics statement

The studies involving human participants were reviewed and approved by the Institutional Ethics Committee of Peking University Third Hospital. Written informed consent to participate in this study was provided by the participants' legal guardian/next of kin.

## Author contributions

ZL, YX, and SW conceived the study. YW, LL, YX, and ZL recruited participants. SW, RZ, XL, YG, and ND collected samples. SW wrote the manuscript and prepared the figures. All authors provided critical intellectual content and approved the final manuscript.

## Conflict of interest

The authors declare that the research was conducted in the absence of any commercial or financial relationships that could be construed as a potential conflict of interest.

## Publisher's note

All claims expressed in this article are solely those of the authors and do not necessarily represent those of their affiliated organizations, or those of the publisher, the editors and the reviewers. Any product that may be evaluated in this article, or claim that may be made by its manufacturer, is not guaranteed or endorsed by the publisher.
