# Supplementary material for: Relationship between maternal–infant gut microbiota and infant food allergy
Source: Front Microbiol. 2022 Nov 7;13:933152. doi: 10.3389/fmicb.2022.933152 (PMC9676664; doi:10.3389/fmicb.2022.933152)
Supplement: Supplementary file 1 [file Data_Sheet_1.docx]

Supplementary Material

# Supplementary Figures and Tables

## Supplementary Figure


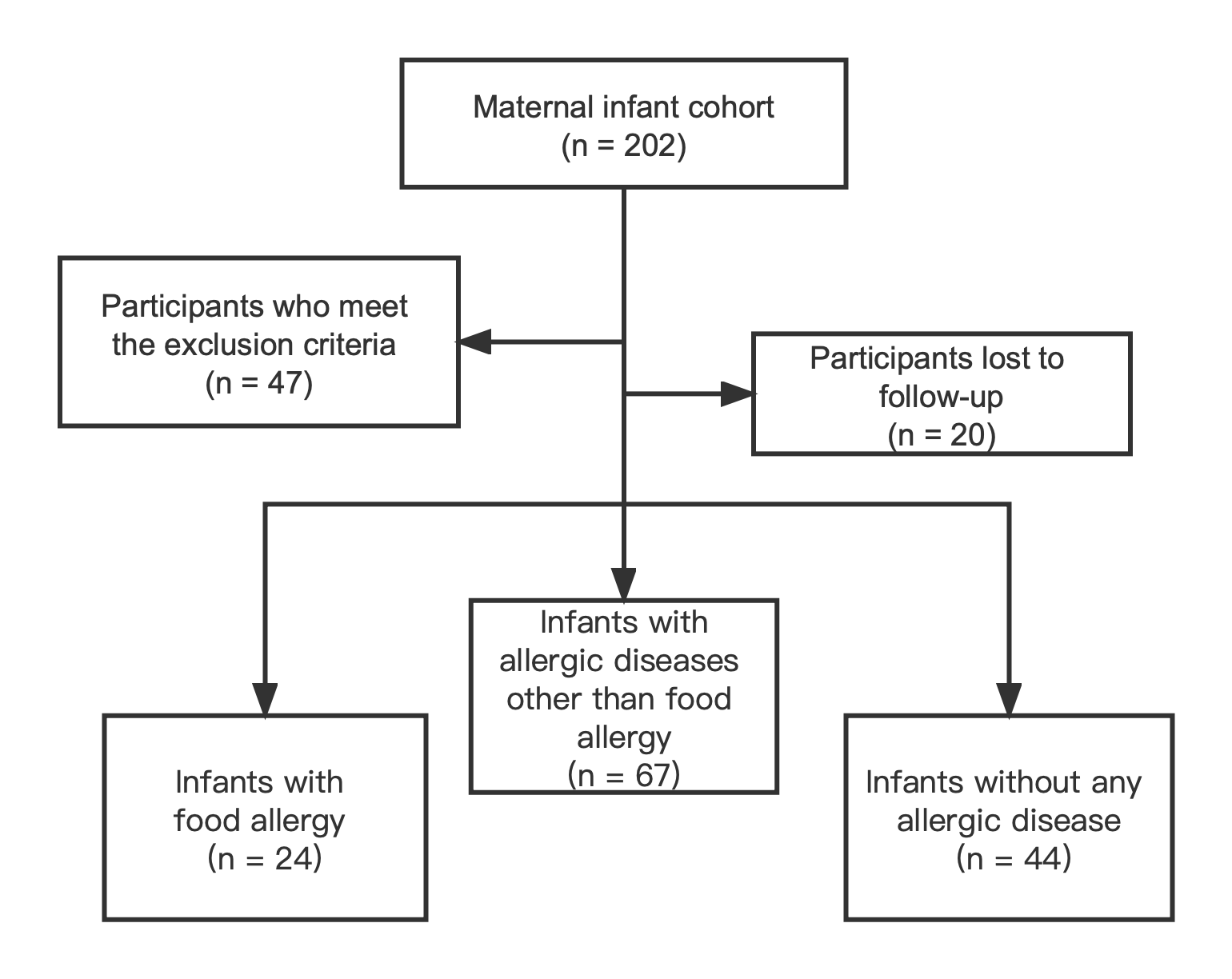


**Supplementary Figure 1.** Flowchart depicting the participant selection for this study.


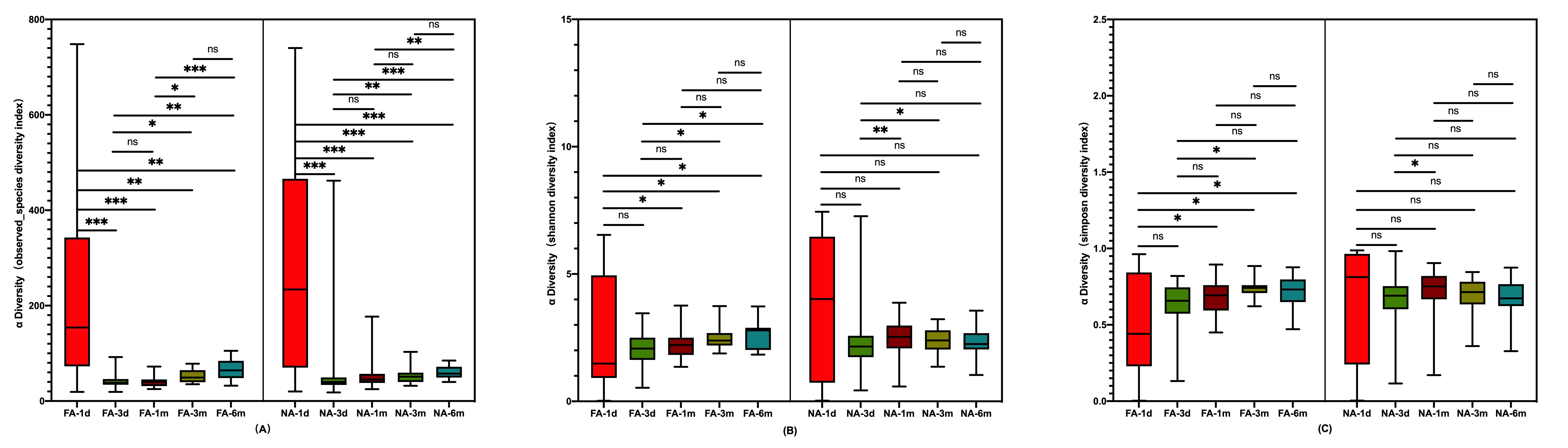


**Supplementary Figure 2.** Differences in variation over time of alpha diversity between the non-allergy (NA) and food allergy (FA) groups using different indices. Observed OTU (**A**) indices reflect species richness, and Shannon (**B**) or Simpson (**C**) indices reflect species diversity. The Kruskal–Wallis test was used to analyze the differences among time groups in the NA and FA groups (**P* < 0.05; ***P* <0.01; ns, not significant).


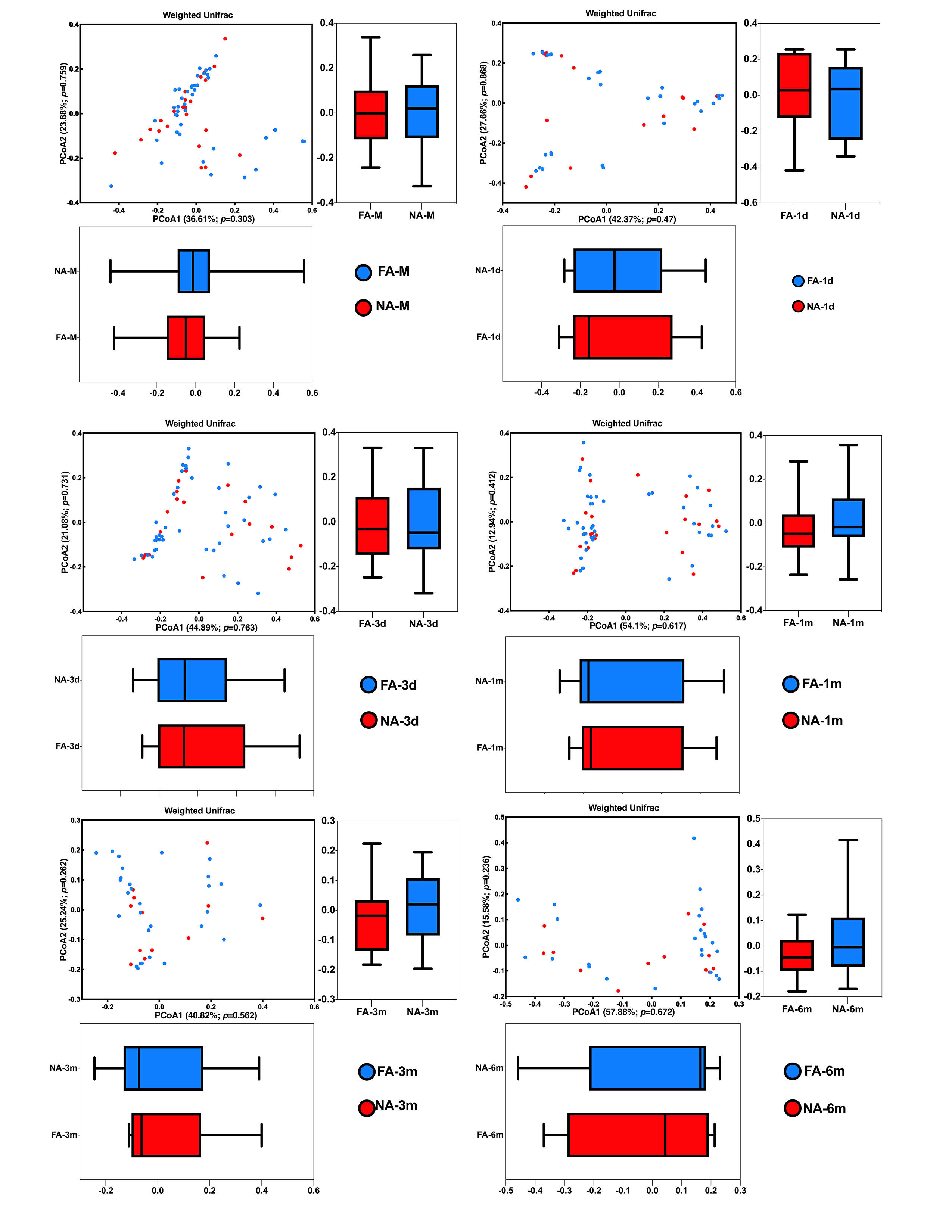


**Supplementary Figure 3.** Differences in the beta diversity of maternal and infant gut microbiota over different periods, as determined using the principal coordinates analysis (PCoA) combined with Adonis analysis. *P* < 0.05 indicates a significant difference.

**1.2 Supplementary Table**

| Group (n) | Maternal samples (n) | Infant samples (n)/Time | | | | |
| --- | --- | --- | --- | --- | --- | --- |
|  |  | Day 1 | Day 3 | 1 month | 3 months | 6 months |
| Non-allergy (n=44) | 42 | 31 | 42 | 41 | 29 | 24 |
| Food allergy (n=24) | 23 | 16 | 22 | 23 | 12 | 13 |
| Total (N=68) | 65 | 47 | 64 | 64 | 41 | 37 |

**Supplementary Table 1.** Number of fecal samples collected at different time points
